# Genotypic variation in the uptake, partitioning and remobilisation of nitrogen during grain-filling in wheat^☆^

**DOI:** 10.1016/j.fcr.2013.10.004

**Published:** 2014-02-01

**Authors:** Peter B. Barraclough, Rafael Lopez-Bellido, Malcolm J. Hawkesford

**Affiliations:** Plant Biology and Crop Science Department, Rothamsted Research, West Common, Harpenden, Hertfordshire AL5 2JQ, UK

**Keywords:** Genotype, Nitrogen, Partitioning, Remobilisation, Uptake, Wheat

## Abstract

Twenty elite varieties of wheat (*Triticum aestivum* L.), primarily winter wheat, were grown with low and high supplies of nitrogen (N) in a field experiment at Rothamsted, southern England, in the season 2004–05. The aim was to quantify genetic variation in the uptake, partitioning and remobilisation of N in individual plant organs at extreme rates of N supply. The biggest contibutor to variation in plant and crop performance was ‘N-rate’ followed by ‘growth stage’ and then ‘genotype’. At both N-rates, there was significant genetic variation in crop performance (grain yield, grain %N, total N-uptake and post-anthesis N-uptake), and in N contents of individual organs at anthesis and maturity, and in N remobilised from individual vegetative organs to the grain during grain-fill. Nitrogen was remobilised from all vegetative organs with very high levels of efficiency by all varieties (80–85%). Stem-N was a major N pool at anthesis probably due to the amounts of soluble N compounds in transit in the vascular system at this time. Despite the genetic variation in N-related plant parameters including stem-N, there were no strong correlations with grain yield and grain %N at a given N-rate. This was probably due to the narrow gene pool employed in this single-season study.

## Highlights

• Twenty elite varieties of wheat grown at 2 N-rates in a field experiment at Rothamsted, UK.
• To quantify genetic variation in uptake, partitioning and remobilisation of N during grain-filling.
• Significant genetic variation in yield and %N, and in N-uptake, organ N-contents and N remobilised from straw to grain.
• N remobilised from vegetative organs with high levels of efficiency (80–85%).
• No strong correlations between N-related plant parameters (e.g. stem-N) and crop performance at a given N-rate.

## 1 Introduction

The yield and quality of wheat grain strongly depend on the availability and uptake of nitrogen (N). High yields of high quality grain can only be achieved with high uptakes of N (Barraclough et al., 2010). A continuing challenge for intensive agriculture is to improve N use efficiency so that yields can be improved or maintained with reduced N inputs. This can be achieved by better recovery of soil and fertiliser-N and by better internal use of N by the plant. In plants, N is needed to grow a leaf canopy for intercepting radiation and for photosynthesis in green tissues. The N requirement for an optimal canopy of winter wheat (for 95% light interception) is 3 g N/m^2^ green area (Sylvester-Bradley and Kindred, 2009), whilst maximum rates of photosynthesis in C_3_ cereals occur at leaf N concentrations above 2 g N/m^2^ green leaf (Sinclair and Horie, 1989). When N supplies are abundant, wheat plants are able to accumulate and store luxury amounts of N (not needed for current growth requirements). This is achieved by producing infertile tillers and by storing N as nitrate, amino acids, amides and soluble proteins in various organs, tissues and organelles of fertile shoots (Millard, 1988). These storage pools have an important function in high input situations where they can improve N uptake efficiency when supplies are abundant (usually pre-anthesis), and buffer dwindling root uptake (usually post-anthesis). It's plausible that stored N could ameliorate the effects of the putative ‘self-destruct’ hypothesis of Sinclair and De Wit (1975) in which they surmised leaf N remobilised to grain was responsible for inducing leaf senescence and hence reducing starch yield. However, Jenner et al. (1991) have disputed that leaf senescence is induced by the N demands of filling grains. Despite the continuing uncertainty over this hypothesis, it seems reasonable to suppose that remobilisation of stored N in preference to photosynthetic N from vegetative tissues would help delay leaf senescence (better yield) whilst meeting the needs for high grain protein (better quality). It would seem that storing N in high input situations is a desirable trait for both bread and feed wheat. However, there is little detailed information in the literature on where (which plant part) and how much N is present in individual plant organs particularly in relation to genetic variation in modern wheat varieties.

Many studies have reported on ‘N-remobilisation during grain-filling’ in wheat, but this has invariably been at a coarse level involving remobilisation from ‘straw’ (that is all the vegetative tissues pooled together) to grain. Examples include: Knowles and Watkin (1931), Spratt and Gasser (1970), Austin et al. (1977), Gregory et al. (1979), Simpson et al. (1983), Cox et al. (1986), Van Sanford and MacKown (1987), Barbottin et al. (2005), Gaju et al. (2011). Very few studies have provided a complete ‘N audit’ during grain filling of wheat; that is how overall crop uptake was partitioned to individual organs and subsequently remobilised to grain. Recent papers have given a more detailed breakdown of N dynamics in one or two varieties of wheat. For example, Bertheloot et al. (2008) monitored the spatio-temporal distribution of N post-anthesis in all organs of the main stems of two wheat varieties. Whilst Pask et al. (2012) measured N in (pooled) leaf laminae, sheaths and stems, and in ears of fertile shoots of a single wheat variety at anthesis and maturity. In addition, Pask attempted to partition the N in individual organs into functional pools – ‘structural’, ‘photosynthetic’ and ‘reserve’.

The aim of the present study was to quantify genetic variation in the uptake, partitioning and remobilisation of N from vegetative organs to grain in a selection of wheat varieties, i.e. to provide a benchmark audit of wheat N relations. Twenty elite wheat varieties were grown at two extreme rates of N in a field experiment at Rothamsted, southern England, in the season 2004–05 and the plants subjected to a full N audit. The experiment was part of a larger series of trials conducted at Rothamsted in a 5-year period (2004–08) specifically designed to study the genetic and environmental variation in N-use efficiency in winter wheat (Barraclough et al., 2010).

## 2 Materials and methods

### 2.1 Site and weather

Rothamsted is located in southern England (latitude 52° N, longitude 1° W). The soil is a flinty, silt clay loam (25% clay) overlying clay with flints (50% clay) designated as ‘Batcombe Series’ in the UK Soil Classification, ‘Aquic Paleudalf’ in the USDA system and ‘Chromic Luvisol’ in the FAO system (Avery and Catt, 1995). In July, the mean maximum temperature at Rothamsted is 21 °C with 190 h of sunshine and a mean daily solar radiation of 15.66 MJ/m^2^. Annual rainfall is typically 700 mm which is spread evenly over the year. In the period March–August 2005, total rainfall was 295 mm compared with the 30-year average of 314 mm. In the same period, mean daily solar radiation was 14.44 MJ/m^2^.

### 2.2 Husbandry

The trial was conducted on Fosters field in the season 2004–05. Twenty winter wheat varieties were sown on 12 October 2004 following winter oats. Plot size was 3 m × 16 m. The wheat was precision-drilled in 12.5 cm rows at a seed rate of 350 m^−2^. Available soil P, K and Mg were at ‘Index 2’ which is non-limiting to yield (MAFF, 2000). The site was top-dressed with 20 kg S/ha as potassium sulphate in March. Crops were given growth regulator and protected against weeds, pests and diseases as required.

### 2.3 Nitrogen fertiliser rates

Nitrogen fertiliser, as ammonium nitrate prills, was applied as a top-dressing at rates of 0 (N0) and 200 (N200) kg N/ha in a 2-way split in mid-March (50 kg N/ha at GS 24) and mid-April (150 kg N/ha at GS 31). Growth stage (GS) refers to Zadoks et al. (1974). Under UK conditions, N0 (plus 30 kg N/ha of N-min measured in the soil profile in February and any soil N mineralised during the season) would be considered deficient, and N200 sufficient for average yields (8–10 t/ha).

### 2.4 Varieties

Twenty elite varieties of wheat (*Triticum aestivum* L.) were grown (Table 1). All varieties were of the winter habit except *cv*. Paragon which is a spring variety (but for this trial was sown in the autumn). The varieties represented a relatively narrow subset of elite genetic material with all but *cv*. Maris Widgeon carrying dwarfing genes. There were 3 varieties from Germany (Batis, Monopol and Sokrates). The remaining 15 varieties had short-straw and appeared on the UK Recommended List in the period 1979–2004 (HGCA, 2010). The UK varieties spanned the quality spectrum from ‘bread’ to ‘feed’ wheat as classified by the National Association of British and Irish Millers (NABIM, 2009). NABIM Group 1 comprises hard wheat with consistently good bread-making properties, Group 2 has bread-making potential in some seasons, Group 3 includes soft varieties suitable for making biscuits and cakes, and wheat in Group 4 is generally only suitable for animal feed.

### 2.5 Experimental design and statistical analysis

The 20 varieties at 2 N-rates were arranged in 3 fully randomised blocks (120 plots). Data were analysed by analysis of variance (ANOVA) using Genstat Release 13.1 (Genstat, 2010). Least significant differences (LSD) are reported at the 5% level of confidence (probably significant) (*P < 0.05) together with the degrees of freedom (df).

### 2.6 Soil N-min

Six soil cores per block were taken to 90 cm depth in February 2005, before fertilizer was applied, to determine the mineral-N status of the site (NO_3_-N and NH_4_-N). The cores were taken with a ‘Hydro Soil Sampler’ fitted with a 3 cm diameter semi-cylindrical auger. Duplicate cores were taken at 3 random positions across each block. The cores were split into 3 depth sections, 0–30, 30–60 and 60–90 cm and the mineral-N extracted by shaking 40 g of fresh soil with 100 ml of 2 M KCl for 2 h. The slurry was allowed to settle for 30 minutes and then filtered (Whatman No.1). The solution was analysed for nitrate-N and ammonium-N with a ‘Skalar San Plus’ analyser. Concentrations in units of ppm in the extracted solution were converted to field units of kg N/ha by assuming a standard value of 1.5 g/cm^3^ for the bulk density of the soil (Avery and Bullock, 1969). No assessments were made of soil N mineralised during the growing season.

### 2.7 Crop and plant measurements

All plots were combine-harvested at grain maturity (GS 92) on 11 August 2005 by cutting a 2 m × 10 m swath from the centre of each plot. Grain yield and grain %N were determined on sub-samples from the combine which were oven-dried overnight at 80 °C. Hand-cuts for shoot growth, N-uptake, ear number and grain and straw yield were made on 3 occasions – 18 April (stem-elongation, GS 31), 8–16 June depending on variety (anthesis, GS 65), and 8–9 August (maturity, GS 92). For the hand-harvests, a 1 m^2^ quadrat (16 rows × 0.5 m) was cut at ground level from each plot. Additionally at GS 65 and GS 92, sub-samples consisting of 20 of the largest fertile shoots were taken for breakdown into component organs. It is likely that most of these shoots were main shoots, but as the main shoots were not tagged, it is possible that some of them were first primary tillers (Zadoks et al., 1974). For convenience, this subsample of 20 large shoots will subsequently be referred to as ‘fertile shoots’. The fertile shoots were broken down into their component organs: leaf-1 (flag leaf lamina), leaf-2, leaf-3, leaf-R (all remaining leaf laminae), leaf sheaths (combined), true stem including peduncle, and ear (further separated into grain and chaff (rachis, palea, lemma, glume and awn) at GS 92). Plant material was oven-dried overnight at 80 °C for determination of dry weight and then milled for determination of N concentration by the Dumas combustion method (Dumas, 1831) using a ‘Leco N’ analyser.

**Fig. 1:**
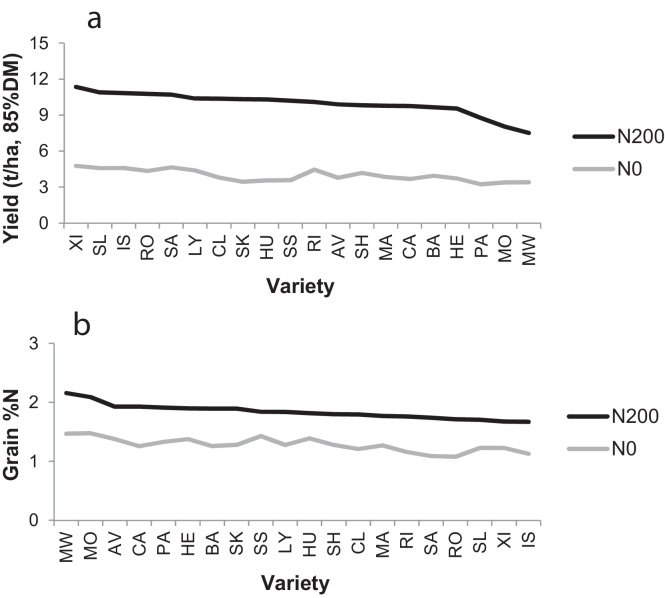
Combine grain yield and grain quality (%N) of 20 wheat varieties at two N-rates ranked on ‘N200’: (a) grain yield (t/ha, 85%DM); (b) grain %N (in DM). LSD (5%): (a) yield – N0 0.92 (38 df), N200 0.78 (37 df), ALL 0.84 (77 df); (b) grain %N – N0 0.07 (38 df), N200 0.12 (38 df), ALL 0.10 (78 df).

**Fig. 2:**
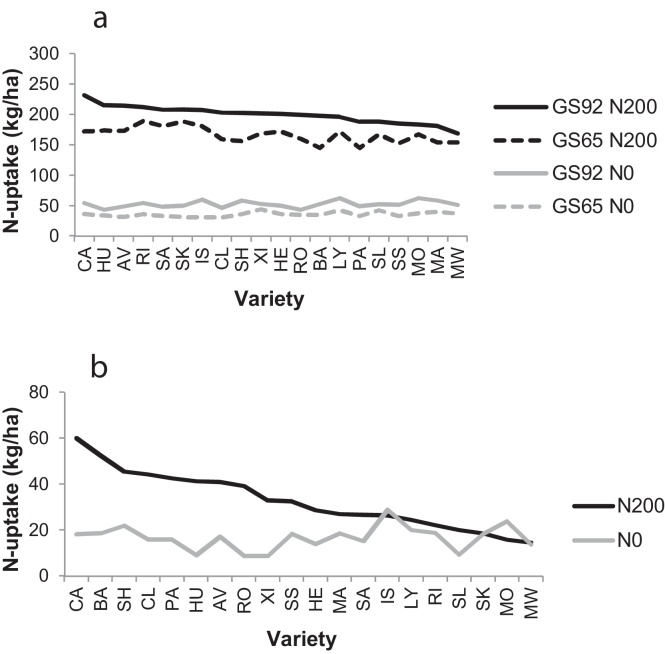
Shoot N-uptake (kg/ha) from hand-cuts at anthesis and maturity ranked on ‘GS92-N200’. LSD (5%): (a) total shoot N-uptake – GS65-N0 13.1 (38 df), GS92-N0 14.2 (35 df), GS65-N200 52.8 (37 df), GS92-N200 37.2 (36 df), ALL 32.8 (152 df); (b) post-anthesis shoot N-uptake – N0 14.2 (35 df), N200 61.2 (36 df), ALL 43.5 (73 df).

**Fig. 3:**
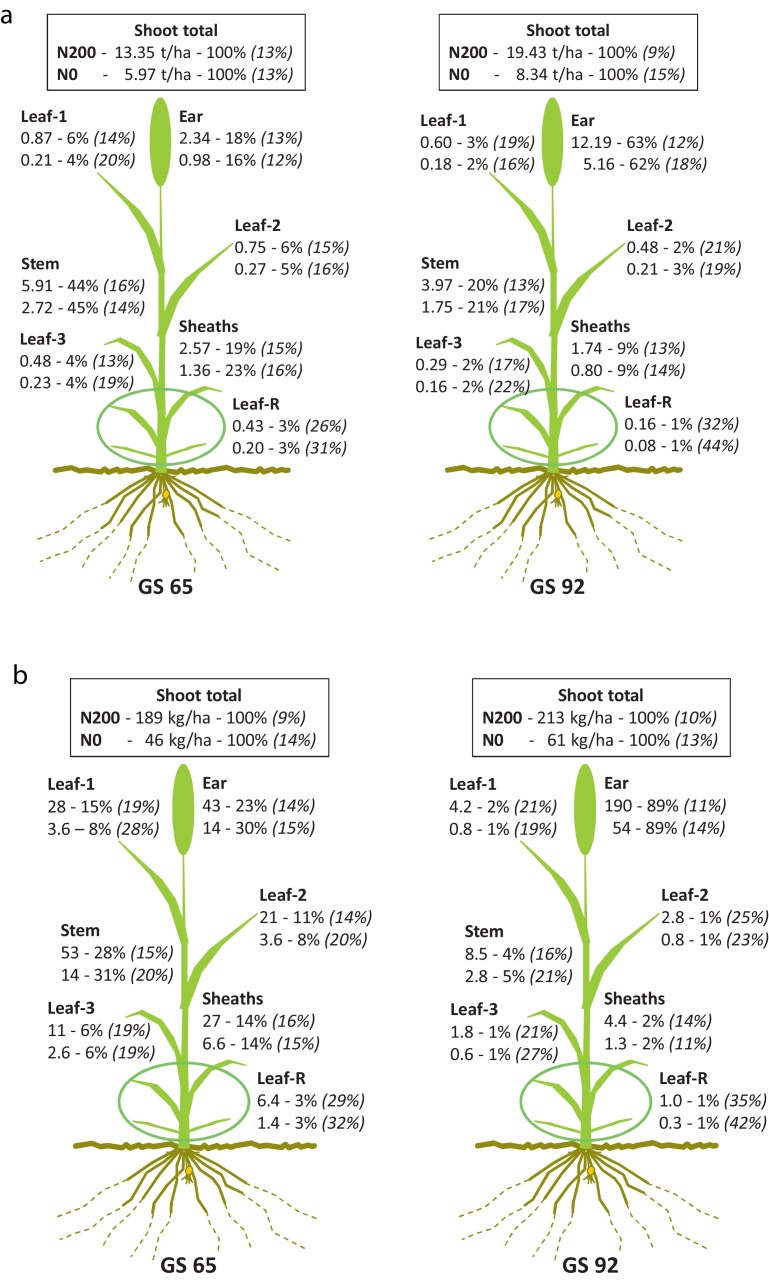
Partitioning of (a) dry matter (t/ha, 100% DM), and (b) nitrogen (kg/ha), in absolute and relative (%) terms, in fertile wheat shoots at anthesis (GS65) and maturity (GS92) at N200 (upper values) and N0 (lower values). Mean of 20 varieties with %CV in italics.

**Fig. 4:**
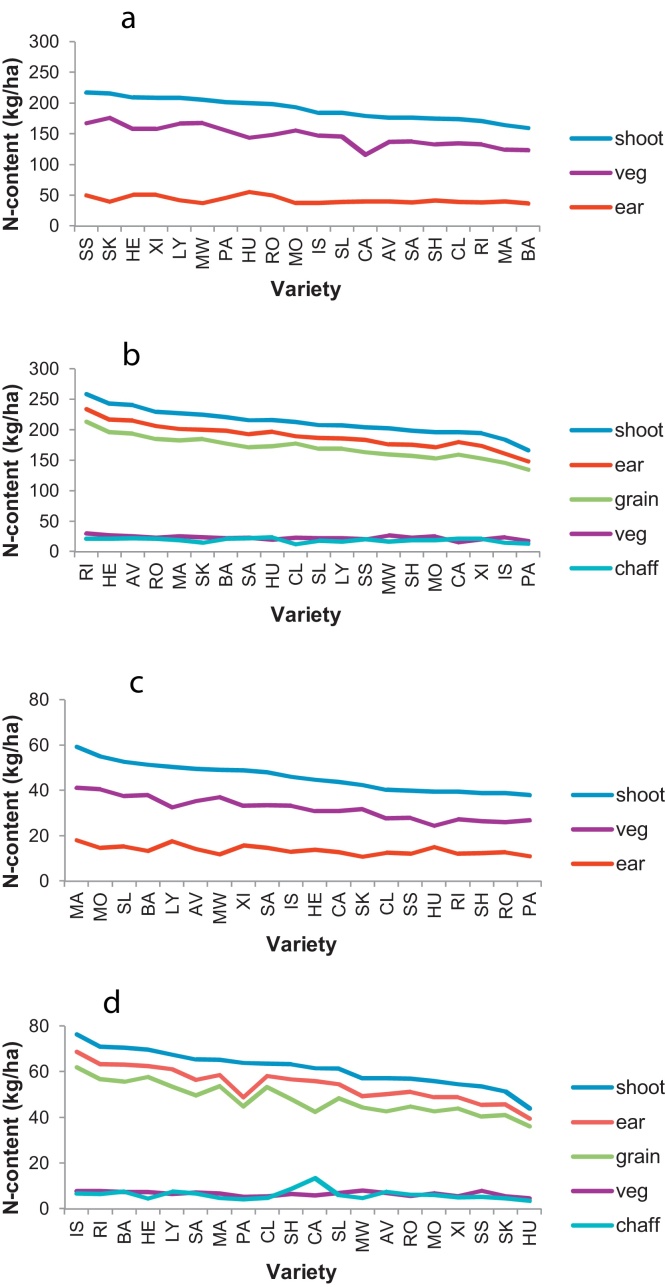
N-content (kg/ha) of vegetative and reproductive organs of fertile wheat shoots ranked on ‘shoot-N’ (veg + ear). LSD (5%) for 34–38 df in brackets: (a) GS65-N200 – shoot (51), vegetative (41), ear (11); (b) GS92-N200 – shoot (52), ear (52), grain (48), vegetative (6.7), chaff (7.7); (c) GS65-N0 – shoot (16), vegetative (12), ear (4.5); (d) GS92-N0 – shoot (20), ear (17), grain (17), vegetative (2.3), chaff (6.5).

**Fig. 5:**
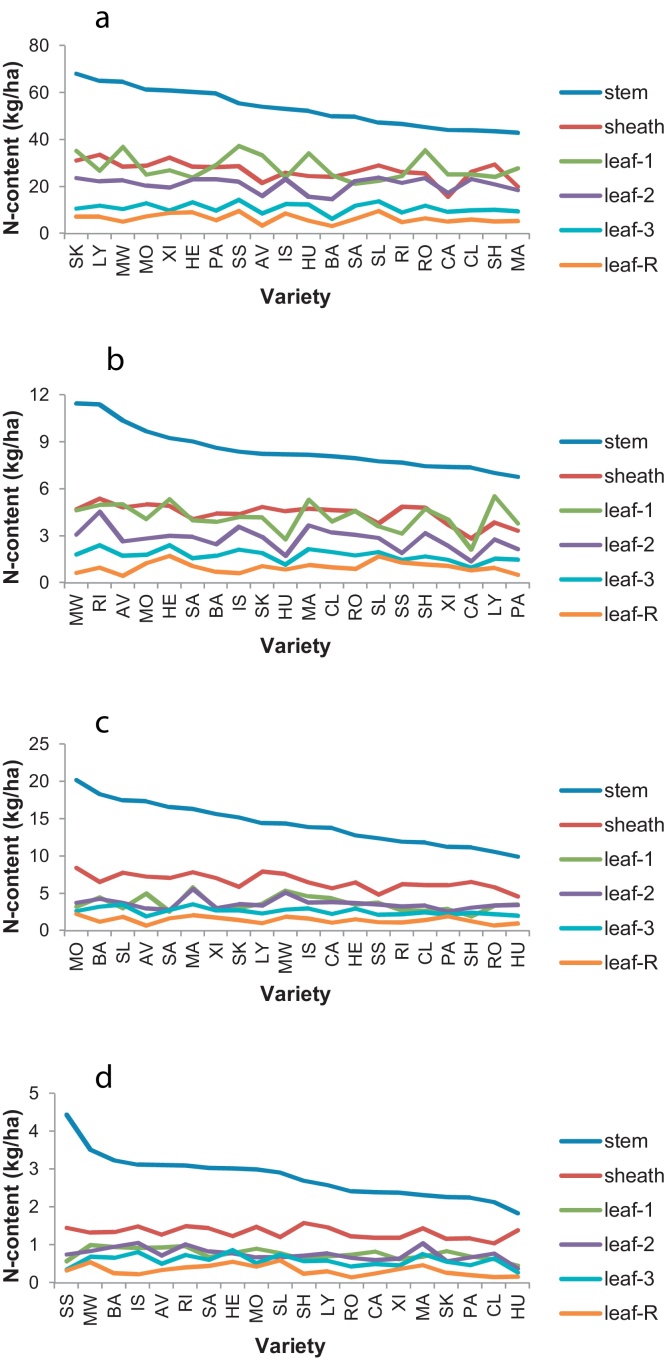
N-content (kg/ha) of individual vegetative organs of fertile wheat shoots ranked on ‘stem-N’. LSD (5%) for 35–38 df in brackets: (a) GS65-N200 – stem (21), sheath (10), leaf-1 (11), leaf-2 (7.9), leaf-3 (3.6), leaf-R (2.4); (b) GS92-N200 – stem (2.8), sheath (1.7), leaf-1 (2.0), leaf-2 (1.1), leaf-3 (0.7), leaf-R (0.8); (c) GS65-N0 – stem (7.6), sheath (2.3), leaf-1 (1.9), leaf-2 (1.8), leaf-3 (1.2), leaf-R (0.9); (d) GS92-N0 – stem (1.1), sheath (0.71), leaf-1 (0.47), leaf-2 (0.34), leaf-3 (0.27), leaf-R (0.26).

**Fig. 6:**
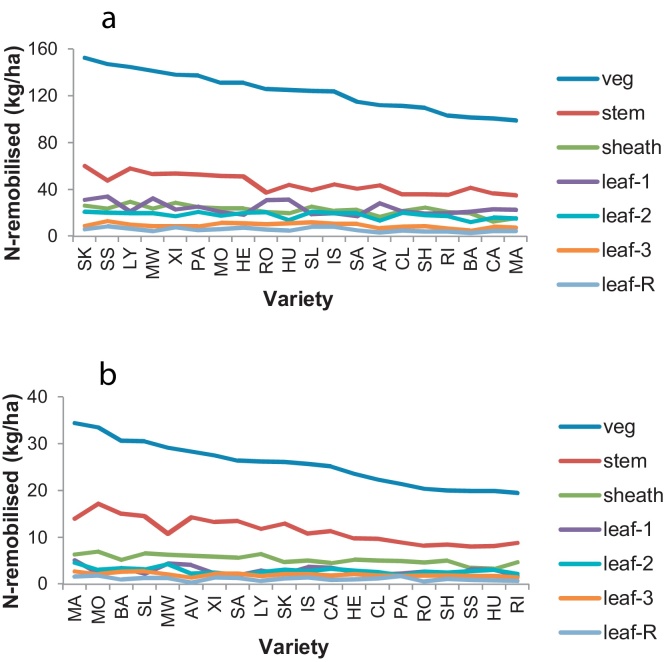
N-remobilised to grain (kg/ha) from vegetative organs of fertile wheat shoots during grain filling (GS65 minus GS92) ranked on ‘vegetative-N’. LSD (5%) for 33–37 df in brackets: (a) N200 – all-vegetative (41), stem (21), sheath (10), leaf-1 (11), leaf-2 (7.8), leaf-3 (3.7), leaf-R (2.7); (b) N0 – all-vegetative (12), stem (7.6), sheath (2.3), leaf-1 (1.8), leaf-2 (1.9), leaf-3 (1.3), leaf-R (0.7).

For the subsample of fertile shoots, nitrogen contents in individual plant organs were measured in units of ‘mg N per organ’. These were scaled-up to field units of ‘kg/ha’ using ear counts made at GS65 and GS92 to allow direct comparison with the crop-scale results shown in Figs. 1 and 2. Ear numbers averaged 296 and 310 ears/m^2^ at N0, and 495 and 507 ears/m^2^ at N200, at GS65 and GS92, respectively. Accordingly, dividing the organ values shown in Figs. 3–6 (in kg/ha) by 3 and 5, at N0 and N200, respectively, will give a rough conversion back to the original units of ‘mg/organ’.

## 3 Results

### 3.1 Crop-scale performance (comprising all shoots)

Combine grain yields, averaged over all varieties, were 3.97 and 9.95 t/ha (85% DM) at N0 and N200, respectively (Fig. 1a). The highest yielding variety at both N-rates was the modern, short-straw bread-wheat Xi19 (4.78 and 11.36 t/ha), and the lowest yielding was the old, tall Maris Widgeon (7.53 t/ha) at N200, and the spring variety Paragon (3.25 t/ha) at N0. Three varieties at N200, Paragon (spring), Monopol (German) and Maris Widgeon (tall) had yields below the declining trend-line, but this was not caused by lodging. Combine grain %N, averaged over all varieties, were 1.28% (varietal range 1.08–1.48%) and 1.84% (varietal range 1.67–2.16%) at N0 and N200, respectively (Fig. 1b). The ranking of grain %N followed the well-known inverse relationship between yield and quality, with low-yielding Maris Widgeon performing best on quality and high-yielding Xi19 poorly. None of the varieties, even at N200, attained 2.3% N in the grain, which is the milling standard for bread wheat in the UK (13% protein). Grain yields were also determined in a final hand-cut at GS 92. Hand-yields were 3% and 8% greater than the combine-yields at N0 and N200, respectively. Straw yields were only determined in the hand-cut not in the combine-cut. Grain harvest index (GHI), the ratio of dry matter in the grain to that in the grain plus straw and chaff, averaged 47.4% (varietal range 40.2–53.3%) and 48.8% (varietal range 39.0–53.2%) at N0 and N200, respectively.

Statistical significance of yield differences (and all other measured and derived N parameters) was assessed by ANOVA for a fully randomised block design. By virtue of the extreme N-rates chosen, ‘N-rate’ (N) was by far the largest contributor to variation and differences were invariably significant to 5% or better. ‘Growth Stage’ (GS) also made a significant contribution to variation. The main thrust of this work was differences due to ‘Genotype’ (G) or ‘Variety’, so most detailed statistics have been presented in relation to this factor. The contribution of interactions such as ‘N × G’ and ‘GS × G’ to the variation was usually small and not significant (Figs. 4–6).

Shoot growth (all shoots) measured in the hand-cuts at GS 31, 65 and 92 averaged 0.82, 5.34 and 7.31 t/ha (100% DM), respectively, at N0; and 1.47, 12.70 and 18.68 t/ha, respectively, at N200. Total shoot N-uptakes determined from these cuts averaged 18, 36 and 52 kg/ha, respectively, at N0; and 43, 167, and 199 kg/ha, respectively, at N200. N-uptake at anthesis was therefore 69% and 84% of that at final harvest at N0 and N200, respectively. The final average N-uptake at N0, 52 kg/ha, was just 4 kg/ha more than was present in the crop at GS 31 (18 kg/ha) plus that in the soil in February (30 kg/ha); so very little net mineralisation or aerial deposition of N occurred during the spring and summer months. Soil N-min in February averaged 30 kg/ha, but varied greatly across the field with values for individual cores in the range 11–57 kg/ha. Nitrogen Harvest Index (NHI), the ratio of N in grain to that in the grain plus straw plus chaff, averaged 83.2% (varietal range 77.6–86.3%) and 83.4% (varietal range 71.9–85.5%) at N0 and N200, respectively.

Total shoot N-uptake by individual varieties at anthesis and maturity (hand-cuts of all shoots) is shown in Fig. 2a. At anthesis, N-uptake averaged 36 kg/ha at N0 (range 31–44 kg/ha), and 167 kg/ha at N200 (range 145–190 kg/ha). By maturity, N-uptake averaged 52 kg/ha at N0 (range 43–63 kg/ha), and 199 kg/ha at N200 (range 169–232 kg/ha). Despite the large varietal range in uptakes, variation between replicates was substantial leading to large LSDs, and very few varietal differences in Fig. 2a were statistically significant. Differences due to ‘N-rate’ and ‘GS’ were significant, but the interactions ‘N × G’ and ‘GS × G’ were not significant. There was a similarly large varietal range in post-anthesis N-uptakes which averaged 17 kg/ha at N0 (varietal range 9–29 kg/ha), and 33 kg/ha at N200 (varietal range 15–60 kg/ha) (Fig. 2b). As with total uptakes, differences due to ‘N-rate’ were significant, but those due to ‘G’ and ‘N × G’ were not significant. Thus the post-anthesis uptake of 60 kg/ha for Cadenza was not significantly different from the 15 kg/ha for Maris Widgeon.

### 3.2 Plant-scale performance (comprising the largest fertile shoots only)

#### 3.2.1 Average performance of all varieties

The partitioning of dry matter (DM) and nitrogen (N) in individual organs (leaf blades, sheaths, stems and ears) of the largest fertile shoots at GS65 (anthesis) and GS92 (maturity), *averaged over all 20 varieties*, is shown in Fig. 3.

Total shoot dry matter at anthesis, averaged over all varieties, was 13.35 t/ha at N200 (Fig. 3a). This was distributed in the order: stem (44%) > sheaths (19%) > ear (18%) > leaf-1 (6%) = leaf-2 (6%) > leaf-3 (4%) > leaf-R (3%). At N0, total shoot dry matter was 5.97 t/ha, and was similarly distributed as at N200. By maturity, total shoot dry matter had increased to 19.43 t/ha at N200. This was distributed in the order: ear (63%) (49% grain, 14% chaff) > stem (20%) > sheaths (9%) > leaves (1–3%). At N0, total shoot dry matter was 8.34 t/ha, and was almost identically distributed as at N200. All vegetative organs lost weight between anthesis and maturity.

Total shoot N-uptake at anthesis, averaged over all varieties, was 189 kg/ha at N200 (Fig. 3b). This was distributed in the order: stem (28%) > ear (23%) > leaf-1 (15%) > sheaths (14%) > leaf-2 (11%) > leaf-3 (6%) > leaf-R (3%). At N0, total N-uptake was 46 kg/ha, and was similarly distributed as at N200, although more was present in the ear (30%) and less in leaf-1 (8%). By maturity, total N-uptake had increased to 213 kg/ha at N200 which was distributed in the order: ear (89%) (80% grain, 9% chaff) > stem (4%) > leaf-1 (2%) > sheaths (2%) > leaf-2 = leaf-3 = leaf-R (1% each). At N0, total N-uptake was 61 kg/ha, and was distributed as at N200. Most N was present in the leaf blades (collectively), but individually the stem was the biggest pool of vegetative N.

Nitrogen was remobilised to the ears from all vegetative organs during grain filling. At N200, the vegetative organs transferred 84% of their N to the ears (124 out of 147 kg/ha). The transfers (kg/ha) were in the order: stems (45) > leaf-1 (24) > sheaths (22) > leaf-2 (18) > leaf-3 (9) > leaf-R (6). At N0, the vegetative organs transferred 79% of their N to the ears (25.5 out of 32.1 kg/ha). The transfers (kg/ha) were in the order: stems (11.5) > sheaths (5.3) > leaf-1 (2.8) > leaf-2 (2.8) > leaf-3 (2.0) > leaf-R (1.1).

#### 3.2.2 Performance of individual varieties

Varietal rankings for N-contents of vegetative and reproductive organs at anthesis and maturity are shown in Fig. 4. At anthesis, total shoot N contents for different varieties ranged from 38 to 59 kg/ha at N0 (Fig. 4c), and from 160 to 217 kg/ha at N200 (Fig. 4a). Vegetative-N ranged from 24 to 41 kg/ha at N0, and from 116 to 176 kg/ha at N200. There was little difference in ear-N between varieties which averaged 14 and 43 kg/ha at N0 and N200, respectively. By maturity, total shoot N contents ranged from 44 to 76 kg/ha at N0 (Fig. 4d), and from 166 to 259 kg/ha at N200 (Fig. 4b). Vegetative-N and chaff-N averaged 6 kg/ha at N0, and 23 and 19 kg/ha, respectively, at N200.

Varietal rankings for N-contents of individual vegetative organs at anthesis and maturity are shown in Fig. 5. All vegetative organs contributed to the varietal range of N contents at anthesis in the order: stem > sheath > leaf-1 > leaf-2 > leaf-3 > leaf-R (Fig. 5a and c). Stem-N ranged from 9.9 to 20 kg/ha at N0 (Fig. 5c), and from 43 to 68 kg/ha at N200 (Fig. 5a). By maturity, stem-N ranged from 1.8 to 4.4 kg/ha at N0 (Fig. 5d), and from 6.8 to 11.4 kg/ha at N200 (Fig. 5b). N-contents of other organs (sheaths and leaf blades) had declined to less than 1.6 kg/ha at N0, and 5.5 kg/ha at N200 (Fig. 5b and d).

The amount of N remobilised from vegetative organs during grain filling (GS 65 minus GS 92) by different varieties ranged from 20 to 34 kg/ha at N0 (Fig. 6b), and from 99 to 153 kg/ha at N200 (Fig. 6a). This calculation assumed that all post-anthesis N-uptake went directly to the ear. Stems were the dominant contributors to this transfer.

## 4 Discussion and conclusions

The most important single factor influencing plant and crop performance in this study was ‘N supply’ (N) because of the extreme range employed (0 and 200 kg/ha). The influence of ‘genotype’ (G) or ‘variety’, by comparison, was muted. A relatively narrow genetic pool was tested in this study as evidenced by yield ranges of just 1.3 and 1.8 t/ha at N0 and N200, respectively (excluding the three varieties, Paragon (spring), Monopol (German variety) and Maris Widgeon (tall)). Despite this narrow pool, significant genetic variation was evident, at both N rates, in measures of crop performance (i.e. grain yield, grain %N and shoot N-uptake) and plant N status, i.e. N contents of individual organs and N remobilised during grain-filling. The contribution of interactions to the overall variation (‘N × G’ and ‘GS × G’) was usually small and non-significant in all the N parameters measured. A feature of the results was the apparent very large genetic range in some of the measured N parameters. For example, there was a substantial varietal range in post-anthesis N-uptake (Fig. 2b), ranging from 15 kg/ha (Maris Widgeon) to 60 kg/ha (Cadenza). However, this and similarly large differences in other parameters were not statistically significant because of the large variability between replicates. It is a salutary lesson of the need to take large samples and/or adequate replication in such studies to cope with the variability in field-measured N-uptakes.

Most plant N was contained in the leaf blades (when all pooled together), followed by stems (all pooled internodes and peduncle) and then leaf sheaths (all pooled) (Figs. 3 and 5). Stems have long been identified as a major N pool (e.g. Gregory et al., 1979), although in most studies ‘stems’ have included sheaths. In a more recent detailed study, Pask et al. (2012) partitioned the ‘fertile shoots’ of a single wheat variety into four components: leaf lamina, leaf sheaths, stems and ears. They further partitioned the N into three pools: structural (SN), photosynthetic (PN) and reserve (RN). SN was defined as that remaining in the shoots at harvest. A functional pool (FN) comprising SN + PN was fixed at 2 g N/m^2^. RN was calculated as the difference between Total-N (TN) and FN. Pask et al. (2012) found that most RN was in the stem and only remobilised with low efficiency (64.0% at Nil N and 61.6% at optimum N). It is not known why this pool would be poorly remobilised if it was truly ‘luxury-N’. In the present study, all varieties remobilised N from all vegetative organs with high efficiency (not shown). On average, 80% and 85% of vegetative-N was remobilised at N0 and N200, respectively. The present measurements did not extend to partitioning stem-N into functional pools. A reasonable measure of ‘structural-N’, following Pask et al. (2012), would be residual-N at maturity which, on average, for stems was 3 and 9 kg/ha at N0 and N200, respectively. It is likely that most of the stem-N at anthesis was in fact ‘metabolic’ and *in-transit* as amino acids in the vascular system, rather than in ‘stored’ or ‘structural’ forms (Simpson and Dalling, 1981).

**Fig. 7:**
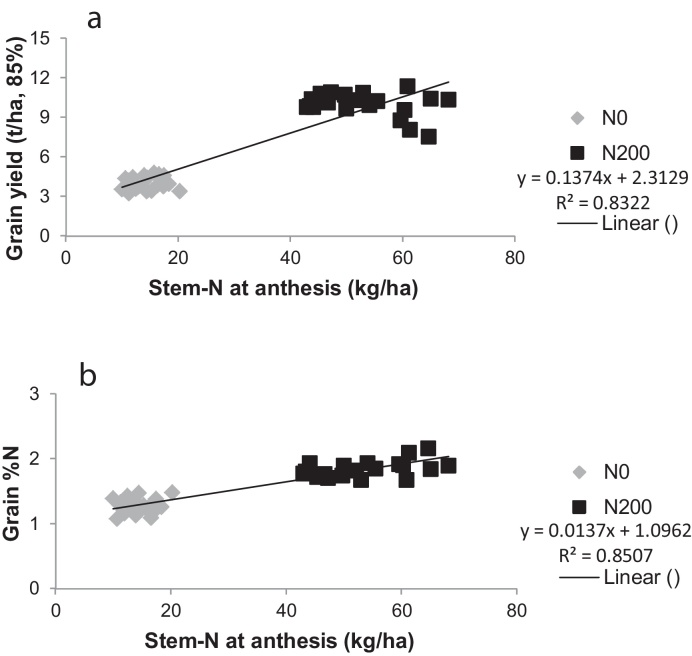
Correlations between stem-N at anthesis and (a) grain yield (t/ha, 85% DM), and (b) grain %N.

Crop performance, i.e. grain yield and grain %N, was well correlated with plant N parameters (e.g. post-anthesis N-uptake, stem-N and vegetative-N at anthesis, and vegetative-N remobilised) when compared over the two N-rates, but there was no correlation at individual N-rates as shown for stem-N at anthesis (Fig. 7). As previously mentioned, this may be due in part to the relatively narrow genetic pool employed in this study. Grain quality has been linked to post-anthesis N-uptake in earlier studies. Monaghan et al. (2001) and Bogard et al. (2010), for example, found that grain protein deviation (the difference from the value predicted by yield) was positively related to post-anthesis uptake. However, Cox et al. (1986) found no relationship between remobilised-N and grain protein concentration. In the present study at N0, grain %N was not correlated with post-anthesis N-uptake, stem-N at anthesis, vegetative-N at anthesis or vegetative-N remobilised, whilst there were weak correlations at N200 (not shown). There were no correlations with yield at either N0 or N200. To take specific examples, the varieties Lynx, Avalon and Paragon had the greatest stem-N contents at anthesis and the greatest amounts of N-remobilised (at N200), but this was not translated into the best yield or grain quality.

This study has provided a benchmark of the uptake, partitioning and remobilisation of N from individual vegetative organs to reproductive organs during grain filling in 20 wheat varieties. But despite there being significant genetic variation in the N contents of different plant parts and its remobilisation to grain, there were no correlations, *at a given N-rate*, with grain yield and quality. This was probably attributable to the narrow genetic pool that was studied in this single-season experiment.

## Figures and Tables

**Table 1:** Wheat varieties showing code, year of release in UK (approx.), NABIM quality group or country of origin (G – Germany).

No.	Variety	Code	Listed	Nabim
1	Avalon	AV	1979	1
2	Batis	BA	*	G
3	Cadenza	CA	1991	2
4	Claire	CL	1999	3
5	Hereward	HE	1991	1
6	Hurley	HU	2003	1
7	Istabraq	IS	2004	4
8	Lynx	LY	1993	2
9	Malacca	MA	1999	1
10	Monopol	MO	*	G
11	Maris W.	MW	1964	1
12	Paragon	PA	1999	1
13	Riband	RI	1989	3
14	Robigus	RO	2003	3
15	Savannah	SA	1998	4
16	Shamrock	SH	1999	1
17	Sokrates	SK	*	G
18	Solstice	SL	2002	1
19	Soissons	SS	1995	2
20	Xi19	XI	2002	1

* Not listed in UK.
